# Fine-tuning the photosynthetic light harvesting apparatus for improved photosynthetic efficiency and biomass yield

**DOI:** 10.1038/s41598-019-49545-8

**Published:** 2019-09-10

**Authors:** N. Friedland, S. Negi, T. Vinogradova-Shah, G. Wu, L. Ma, S. Flynn, T. Kumssa, C.-H. Lee, R. T. Sayre

**Affiliations:** 1 New Mexico Consortium, Los Alamos, NM 87544 USA; 2 Pebble Labs, 100 Entrada Drive, Los Alamos, NM 87544 USA; 3 Department of Molecular Biology, Pusan National University, Busan, 46241 Republic of Korea; 4 University of Nebraska, Lincoln, NE United States

**Keywords:** Biophysics, Antenna complex

## Abstract

Photosynthetic electron transport rates in higher plants and green algae are light-saturated at approximately one quarter of full sunlight intensity. This is due to the large optical cross section of plant light harvesting antenna complexes which capture photons at a rate nearly 10-fold faster than the rate-limiting step in electron transport. As a result, 75% of the light captured at full sunlight intensities is reradiated as heat or fluorescence. Previously, it has been demonstrated that reductions in the optical cross-section of the light-harvesting antenna can lead to substantial improvements in algal photosynthetic rates and biomass yield. By surveying a range of light harvesting antenna sizes achieved by reduction in chlorophyll *b* levels, we have determined that there is an optimal light-harvesting antenna size that results in the greatest whole plant photosynthetic performance. We also uncover a sharp transition point where further reductions or increases in antenna size reduce photosynthetic efficiency, tolerance to light stress, and impact thylakoid membrane architecture. Plants with optimized antenna sizes are shown to perform well not only in controlled greenhouse conditions, but also in the field achieving a 40% increase in biomass yield.

## Introduction

Plants have evolved highly efficient photosynthetic light harvesting antenna that operate with varying energy conversion efficiencies over a wide variety of natural light conditions This flexibility allows for adaptability to both high and low light conditions as well as facilitates competition for light in mixed species consortia. Light-harvesting antenna sizes are restricted, however, in their ability to maximize photosynthetic efficiency in varying light regimes. In plant canopies this leads to a situation in which the rate of photosynthetic electron transport in upper leaves is saturated at high light intensities (full sunlight) resulting in the dissipation of excess captured energy as heat and fluorescence. In contrast, photosynthetic electron transport in shaded regions of the canopy may be light-limited. This imbalance of light availability and utilization between different levels in the plant canopy is a major bottleneck in improving net photosynthesis in monocultures and therefore enhancing crop yields^1^.

At full sunlight intensities, light harvesting antenna complexes capture light at a rate (10 photons per us) which significantly exceeds the rate of down-stream proton-coupled electron transfer reactions (1–10 ms) leading to the dissipation of excess captured energy^2^. One strategy to better couple the rate of light capture to the downstream electron transfer processes while improving light penetration throughout the canopy is to reduce the apparent optical cross-section of the light harvesting antenna complex^3^. This approach has been successfully implemented in algal cultures whose self-shading increases temporally as cultures grow Optimization of light harvesting antenna in algae resulted in a 40% increase in overall biomass yield^4,5^. One means to reduce light harvesting antenna size is to reduce the accumulation of chlorophyll *b* (Chl *b*). Chl *b* accounts for approximately half the Chl in peripheral light harvesting complexes (LHC) and is not present in the photosynthetic reaction centers. The LHC apoproteins which bind Chl and other pigments are made in the cytoplasm, imported into chloroplasts and folded in the presence of the photosynthetic pigments to form stable protein pigment complexes. Reductions in Chl *b* levels have been shown to reduce the stability of the LHC proteins leading to their degradation and reductions in the light harvesting antenna cross section^6^.

The synthesis of Chl *b* is catalyzed by chlorophyllide *a* oxygenase (CAO), a chloroplastic enzyme catalyzing a two-step oxygenation reaction converting chlorophyllide *a* into chlorophyllide *b*^7^. It has been demonstrated that manipulations of CAO levels result in altered Chl *b* content in plants and algae^4,8^. Thus, CAO is a logical target to control the amount of Chl *b* and hence the antenna size.

We have compared photosynthetic and biomass performance characteristics in plants engineered to have a range of reductions in Chl *b* levels and light harvesting antenna size. We show that plants with slightly reduced antenna sizes (Chl *a/b* ratio ~ 5.0) have improved photosynthetic performance and biomass yield compared to wild-type plants (Chl *a/b* ~ 2.5–3.9). In addition, further reductions in this optimal light harvesting antenna size leads to an abrupt drop in photosynthetic efficiency, reduced light stress tolerance, and reduced biomass accumulation. These results indicate that there is a tipping point in antenna size where larger or smaller antenna sizes not only impacts light capture efficiency but light stress tolerance and overall electron transport rates.

## Results

### Transgenic plants demonstrate a range of phenotypes based on the degree of Chl *b* reduction

We chose *Camelina sativa*, a *Brassicaceae* family member, as a test case for antenna modification and phenotypic characterization. *Camelina sativa* has emerged as a promising biofuel feedstock due to its many desirable characteristics, including wide adaptability to different climate conditions, as well as low water, fertilizer and pesticide requirements^9^. *Camelina* is also an excellent model plant for antenna modifications due to its dense vertical leaf canopy architecture.

We hypothesized that there is an optimal range of Chl *a/b* ratios that would produce plants with improved photosynthetic performance. Our assumption was based on the following previous observations: (1) algae with intermediate antenna sizes grow faster than wild-type algae^4^; (2) soybean and tobacco cultivars with reduced Chl *b* levels are higher yielding than wild-type cultivars^10^; and (3) plants completely lacking Chl *b* have very small light harvesting antenna and significantly reduced growth^11^.

**Figure 1:**
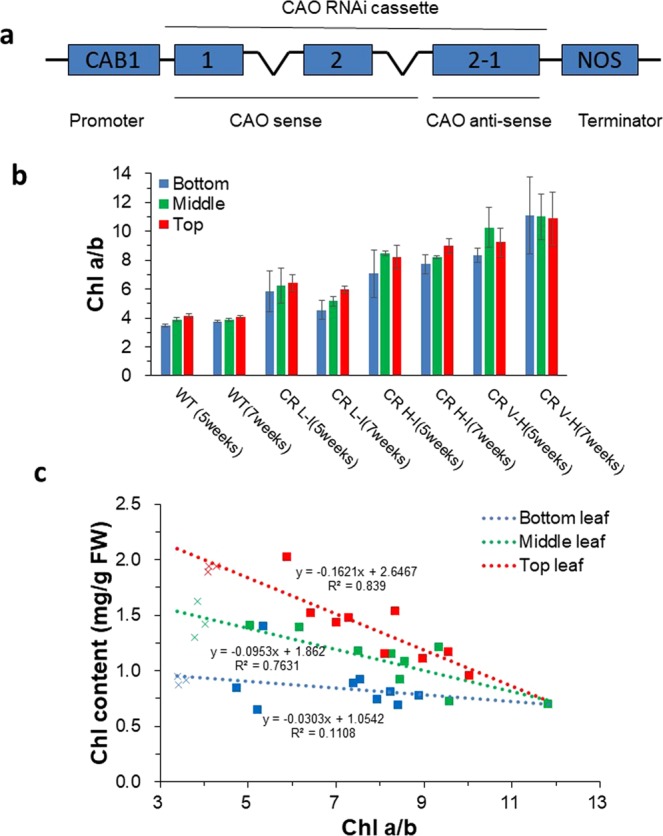
RNAi silencing of CAO gene increases Chl *a/b* ratio in transgenic plants (**a**) A schematic representation of the gene construct used to induce RNAi silencing of the CAO genes in *C. sativa*. Exons are represented by grey-blue boxes and introns by “V”s. (**b**) Chl *a/b* ratio of fully expended non-senescent leaves at the bottom, middle and top position of plants of 5 and 7 week-old age. The results represent the average and SD of three independent experiments (**c**) Total Chl per fresh weight of bottom, middle and top position of leaves of 5 week-old plants with different Chl *a/b* ratios. WT are shown as crosses and CR transgenic plants as squares. Chl content from leaves was determined according to Porra *et al*.^34^.

We engineered transgenic Camelina plants to have altered light harvesting antenna sizes utilizing an RNAi approach to reduce Chl *b* levels by modulating the expression levels of the CAO gene. A genomic hairpin CAO sense/antisense construct spanning the first two exons of the CAO gene and including an intron (hairpin loop region) from *A. thaliana* was expressed under the control of leaf-specific CAB1 promoter (Fig. 1a, Supp Fig. S1a). Transgenic plants, identified by PCR and DNA sequence analysis for presence of the transgene. Confirmed transgenic plants were subsequently screened for altered Chl *a/b* ratios, which ranged from 4 to 19. For subsequent experiments, we focused on three groups of backcrossed CAO-RNAi lines (>T3 generation) covering a range of Chl *a/b* ratios and antenna sizes to investigate the dependence of plant photosynthetic performance on the degree of antenna size reduction.

The transgenic plants were assigned to three different groups according to their Chl *a/b* ratios and growth phenotypes (determined at 3–5 week of age) including: low-intermediate lines (CR L-I) having Chl *a/b* ratios ranging from 4.5 to 6.5, high-intermediate (CR H-I) lines having Chl *a/b* ratios ranging from 6.5 to 8.5, and very high lines (CR V-H) having Chl *a/b* equal to or greater than 8.5.

For each group, we compared Chl *a/b* ratios of fully expanded leaves at the top middle and bottom position of the plant. As shown in Fig. 1b and Supp Table S1, Chl *a/b* ratios were greater towards the top of the canopy and smaller towards the bottom of the canopy for wild-type and transgenic plants reflecting an increase in antenna size from the top to the bottom of the canopy. All transgenic plants had higher Chl *a/b* ratios compared to WT plants at the corresponding leaf positions. This trend was observed for both 5 and 7 week-old plants.

Furthermore, we observed an inverse correlation between Chl *a/b* ratios and total Chl content per gram fresh weight in upper and mid-level leaves, but not lower leaves of transgenic plants. This reduction in Chl content associated with elevated Chl *a/b* ratios was greatest in upper leaves and lowest in the bottom leaves of the canopy (Fig. 1c). In contrast to wild-type, CR L-1, and CR H-1 plants, the Chl content/gram fresh weight of CR V-H leaves at different positions in the canopy was not significantly different suggesting that CR V-H plants had maximal reductions in Chl content at all levels in the plant canopy. In agreement with this interpretation, all leaf positions of seven week old CR V-H plants had similar and very high Chl *a/b* ratios.

For plants with dense canopies, light availability is a major limiting factor for net photosynthetic gain^12^. As shown in Supp Fig. S2a,b, the light transmission though an individual leaf significantly increases with antenna size reduction. Wild-type leaves transmitted 36% less PAR compared to leaves of transgenic plants with a higher Chl *a/b* ratio (CR L-I line) allowing for increased light penetration through the canopy and improved biomass accumulation for CR L-I lines^13^.

### CR L-I plants with moderate reduction in antenna size have increased photosynthetic rates

**Figure 2:**
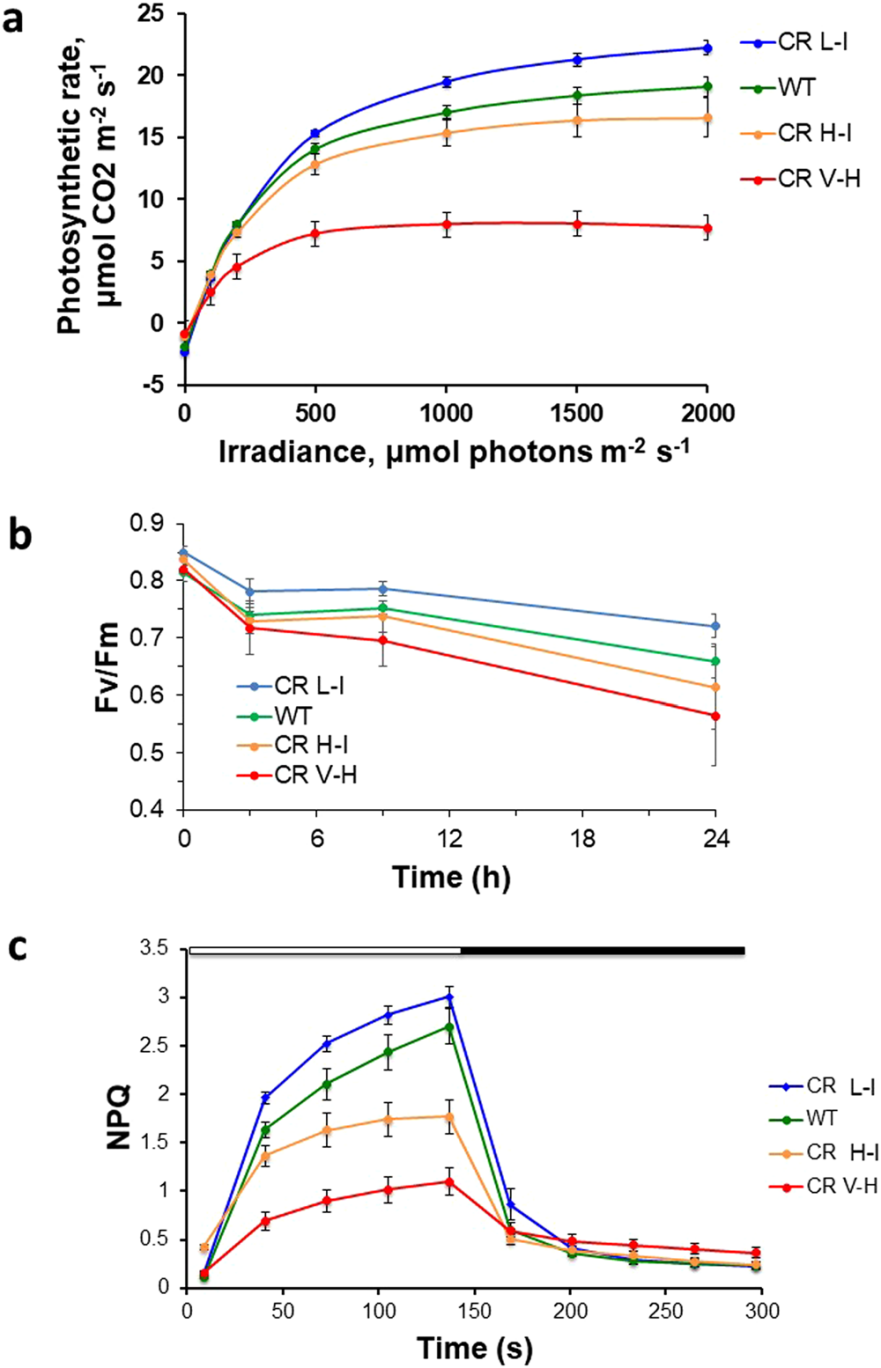
Comparison of photosynthetic traits in WT and transgenic plants. (**a**) Light saturation response curves of photosynthetic CO_2_ fixation rate of WT, CR L-I (Chl *a/b* = 4.5–6.5, CR H-I (Chl *a/b* = 6.5–8.5) and CR V-H (Chl *a/b* 8.5 or above). (**b**) Time-dependent changes in the photochemical efficiency of PSII or Fv/Fm in leaves of WT and the 3 different CR transgenics under high light (HL) stress at 1,000 μmol photons m^−2^ s^−1^. (**c**) Non-photochemical quenching (NPQ) rise and relaxation kinetics. All experiments were done on 3–5 week old plants. Results represent the average and SD of three independent measurements. Chl content was determined according to Porra *et al*.^34^.

To determine the impact of antenna size alterations on photosynthesis, we compared light intensity-dependent rates of CO_2_ fixation (Fig. 2a) in greenhouse grown plants. Compared to WT, CO_2_ fixation rates in CR H-I transgenics were reduced 10–15% compared to wild-type plants at all light intensity ranges tested. Photosynthetic CO_2_ fixation rates were even more severely impaired in CR V-H transgenics having the smallest antenna sizes, consistent with their stunted growth phenotype. Not surprisingly, there was also a slight reduction of photosynthetic rates in the best performing CR L-I line at low light intensities. This is not unexpected since plants with truncated antenna would have less saturated rates of photosynthesis at higher light intensities relative to wild-type plants. Importantly, however, under high light conditions, the areal photosynthesis rates for the CR L-1 plants was 17% greater than WT. Thus, plants with slightly reduced antenna sizes have improved photosynthetic performance under high light conditions relative to WT, while further reductions in antenna size lead to substantial impairments in photosynthetic rates.

At high, saturating light intensities photosystem II electron acceptors can be over reduced due to downstream rate limitations in photosynthetic electron transport. Under these conditions closed photosystem II reactions centers are susceptible to photodamage and turnover of photosystem II complexes. To determine whether alterations in antenna size impacted the sensitivity of photosystem II to light damage, we measured PSII photochemical efficiency (Fv/Fm) following exposure to high light intensities (1,000 μmol photons m^−2^ s^−1^) typically observed in the field. It was anticipated that reductions in antenna size would be directly correlated with sensitivity to photodamage. This was not the case, however. Plants with the smallest light harvesting antenna were more sensitive to high light-induced photodamage than wild-type plants or plants with slightly smaller antenna optimal for photosynthetic efficiency (Fig. 2b). High light sensitivity for CR H-I transgenics was similar to wild type consistent with other photosynthetic performance parameters observed for transgenic plants having optimal Chl *a/b* ratios near 5.

### Non-photochemical quenching in WT and transgenic lines

To further understand the molecular basis for light stress sensitivity in plants with altered light harvesting antenna sizes, we examined non-photochemical quenching (NPQ) processes following light stress. NPQ is an important photoprotection mechanism which reduces the formation of damaging reactive Chl radicals and oxygen species generated under high light stress. NPQ dissipates excess captured energy as heat via the zeaxanthin cycle. Mutants lacking Chl *b*, LHCII proteins, or exhibiting alterations in the topological organization of PSII antenna are known to have substantially reduced capacity to dissipate excess energy by NPQ, demonstrating the intricate involvement of antenna proteins in the NPQ process^14,15^.

We analyzed NPQ kinetics and yield in control (non-stressed) WT and transgenic lines following a 12 hour darkness (night) period. As shown in Fig. 2c, CR L-I and CR H-I transgenic lines exhibited NPQ kinetics similar to WT, but NPQ developed more slowly in the CR V-H line. NPQ relaxation kinetics were also slower in the CR V-H line compared with WT and the other two CR transgenic lines. Short-term NPQ relaxation kinetics corresponds to qE relaxation, which suggests that qE energy quenching mechanism is normal in CR L-I and CR H-I but impaired in CR V-H having the smallest antenna size. These results are consistent with the increase loss in photosynthetic efficiency in transgenics having smaller light harvesting antenna sizes (Fig. 2b). Plants having reduced NPQ capacity would be expected to be more susceptible to photodamage at high light intensities.

### Analysis of thylakoid composition by blue-native gel electrophoresis and sucrose density gradient ultracentrifugation

**Figure 3:**
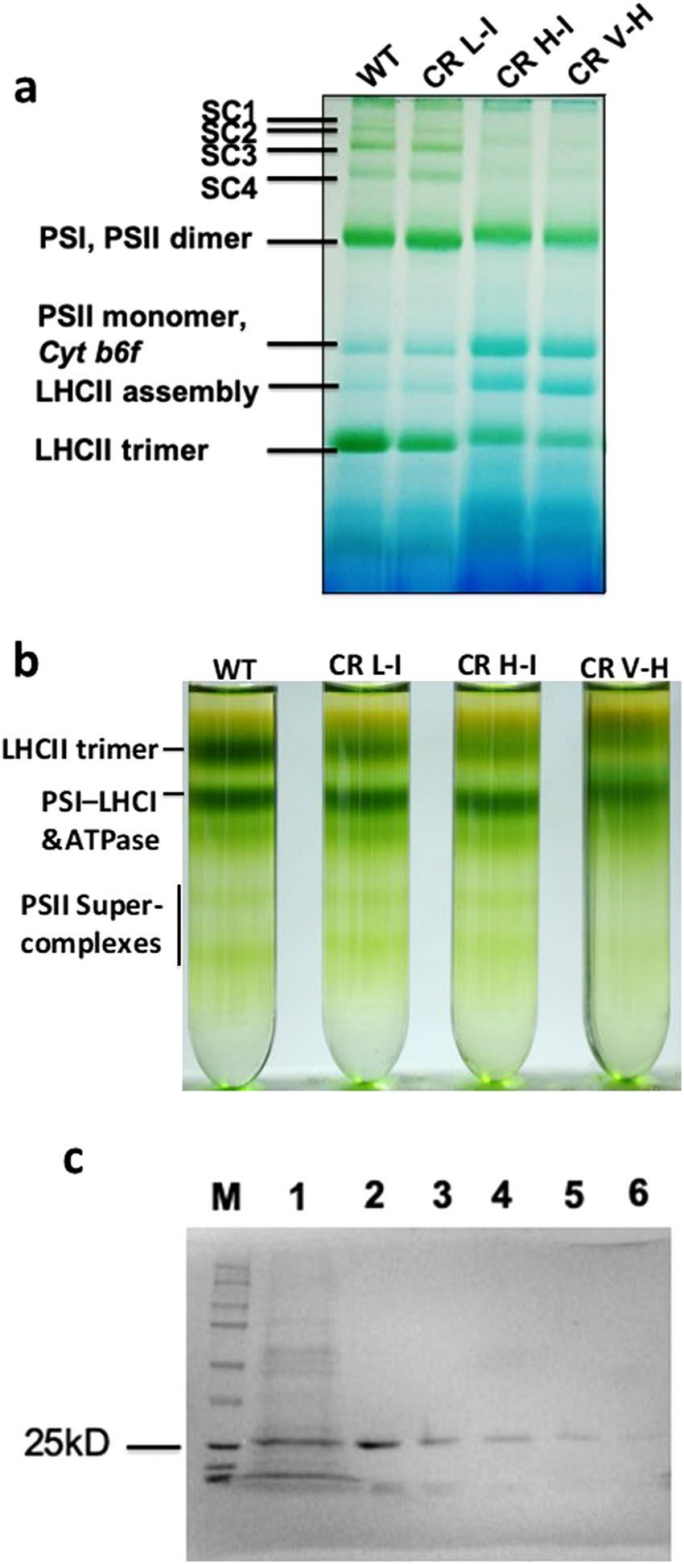
BN-PAGE analysis of PSII supercomplex organization and abundance as a function of Chl *b* abundance. Thylakoid membranes were isolated from WT (Chl *a/b* was 3.4), CR L-I (Chl *a/b* was 4.6), CR H-I (Chl *a/b* was 7.9) and CR V-H (Chl *a/b* was 10.0) lines. (**a**) An aliquot of thylakoid suspension containing 8 μg of Chl was solubilized with final concentration of 1% (w/v) β-DM and was loaded on a 4.5–13.5% BN-PAGE. Identities of the photosystem complexes are given on the left of the panel. The PSII supercomplexes are assigned as follows: SC1 (C_2_S_2_M_2_), SC_2_ (C_2_S_2_M), SC3 (C_2_S_2_) and SC4 (C_2_S). The identification of all other bands was made according to Rantala *et al*.^17^. (**b**) CP complexes isolated by sucrose density gradient ultracentrifugation. Thylakoid membranes were isolated from mixed leaves from 6 plants of each WT (Chl *a/b* was 3.5), and CR L-I (Chl *a/b* was 5.2), CR H-I (Chl *a/b* was 8.0) and CR V-H (Chl *a/b* was 9.8) lines. The identification of all bands was made according to Barera *et al*.^21^. (**c**) SDS-PAGE analysis of LHCII trimer. M-Molecular weight markers, 1-Total thylakoids isolated from WT plants, 2-LHCII trimer excised from BN-gel (Fig. 3a, WT), 3-LHCII trimer fraction from SDGU (WT), 4- LHCII trimer fraction from SDGU (CR L-I), 5- LHCII trimer fraction from SDGU (CR H-I), 6- LHCII trimer fraction from SDGU (CR V-H). Chl content was determined according to Porra *et al*.^34^.

To determine if the decrease in photosynthetic performance in transgenic plants having Chl *a/b* ratios >5 is correlated with dramatic changes in light harvesting protein-complexes, we analyzed the composition and relative abundance of various light harvesting supercomplexes using detergent solubilized thylakoids fractionated on blue native-PAGE (BN-PAGE), a technique commonly used to characterize intact thylakoid membrane-protein complexes^16^. Following supercomplex fractionation on non-denaturing BN-PAGE conditions, we observed 7 major chlorophyll-containing bands (Fig. 3a). The four upper bands on the gel represent various forms of PSII supercomplexes based on routine assignments based on similar analyses carried out using the closely related plant, Arabidopsis^17–20^. For higher plants, the largest molecular weight supercomplex, SC1, consists of a dimeric core (C_2_), two LHCII trimers (S) strongly bound to the complex, and two more trimers, the moderately bound (M) trimers^17–20^. The smaller molecular weight supercomplexes are assigned as C_2_S_2_M (SC2), C_2_S_2_ (SC3), and C_2_S (SC4), based on previous studies^17,18^. We observed that the abundance of supercomlexes containing M-trimers, as well as unattached loosely bound L-trimers (LHCII trimer in Fig. 3a), decreased with increasing Chl *a/b* ratios. The changes in apparent Chl abundance in various supercomplexes between transgenic lines CR L-I to CR H-I lines suggests a significant reduction of higher order supercomplexes, as well as of L-trimers as the peripheral antenna size was reduced with increasing Chl *a/b* ratios.

To further investigate the effects of alterations in Chl *a/b* ratios on the peripheral light harvesting complex observed on the BN-PAGE (Fig. 3a), we detergent solubilized thylakoid membranes and separated the LHCII trimers from the remaining Chl-protein supercomplexes by sucrose density gradient ultracentrifugation (SDGU) (Fig. 3b). To determine the identity of proteins in LHCII trimers separated by SDGU, we analyzed their protein diversity by SDS-PAGE (Supp. Fig. S3). We observed only a single 25kD polypeptide in LHCII trimer bands by Coomassie staining. To determine the relative reductions in LHCII trimer pigment levels, we measured the total Chl in the LHCII bands. We observed that Chl levels in LHCII trimers in the transgenic lines were reduced by 21%, 38% and 54% for the CR L-I, CR H-I and CR V-H lines, respectively, relative to WT. Consistent with previous observations, we observed that the Chl *a/b* ratio (1.56) of WT LHCII trimers was similar to that observed by Barera *et al*.^21^. However the Chl a/b ratios of transgenic lines were substantially greater (2.0 to 4.5) than those of WT reflecting a possible substitution of Chl *a* for Chl *b* and/or a loss of Chl b (Supp Table S2).

### Thylakoid membrane stacking is altered with antenna modification

**Figure 4:**
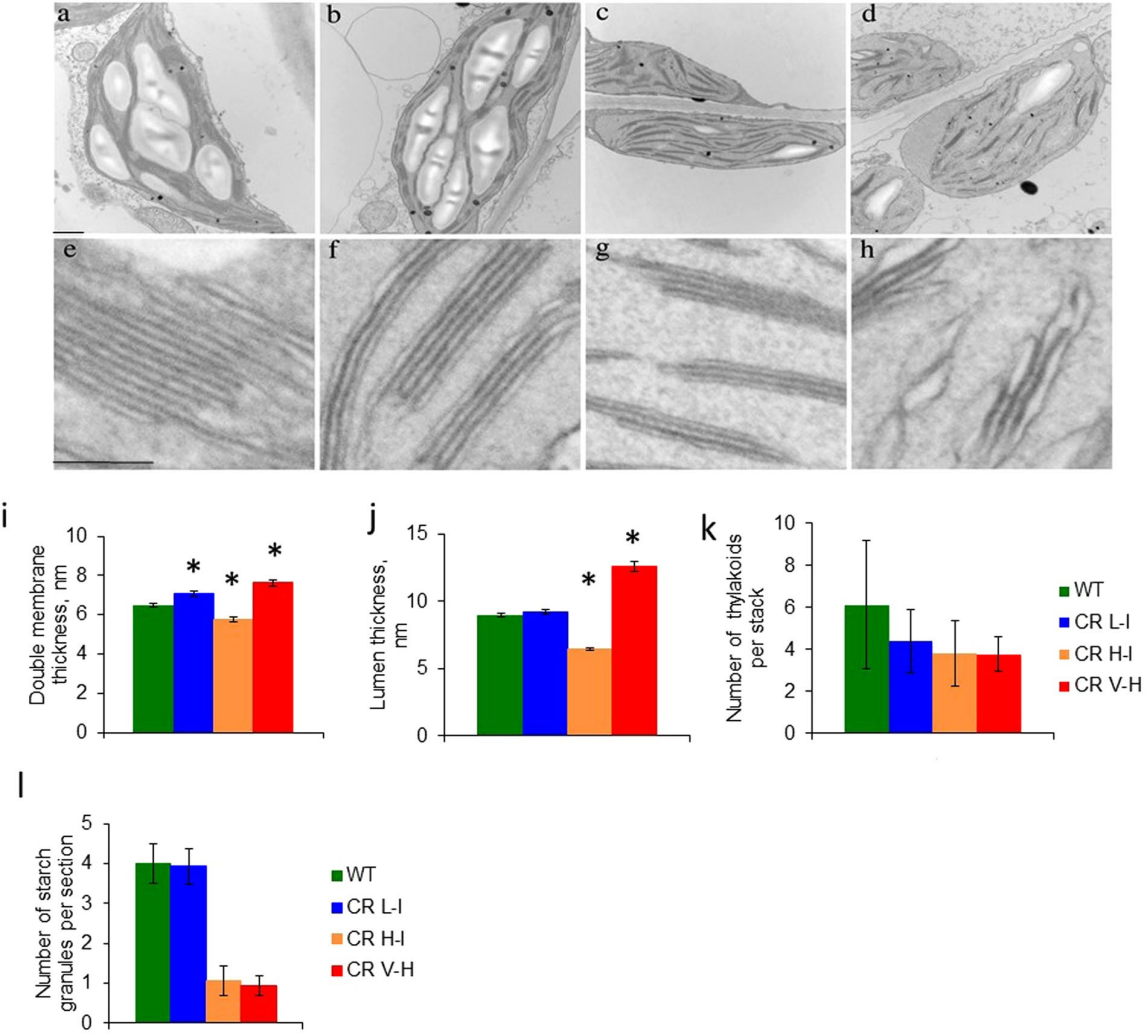
Comparison of thylakoid structure of WT and CR transgenics. (**a**–**h**) Analysis of thylakoid membranes from the wild type and CR transgenic lines by transmission electron microscopy. Leaves from 3-week-old wild-type and CR L-I, CR H-I, and CR V-H plants were directly fixed 3 h after the start of the light phase of the growth photoperiod and prepared for transmission electron microscopy. Chloroplast sections are shown for the wild type (**a,e**), CR L-I (b,f), CR H-I (**c,g**), and CR V-H (**d,h**) plants. Bars in the top and bottom panels = 50 and 100 nm, respectively (**i**). The thickness of thylakoid double membranes in WT and CR L-I, CR H-I, and CR V-H transgenics, (**j**) The thickness of lumen in WT and CR transgenics, (**k**) Amount of thylakoids per granum in wild type and mutants. Average and maximum number of thylakoids per stack are shown **(l**). Amount of starch granules per section in chloroplasts of WT and CR L-I, CR H-I, and CR V-H plants. Values are based on at least 15 independent measurements plus or minus SD.

To determine if the loss of LHCII trimers in transgenic lines impacted membrane architecture, we examined chloroplast thylakoid membrane organization in *C. sativa* WT as well as in the transgenic lines by transmission electron microscopy (TEM). It was previously demonstrated that Chl *b* deficient mutants exhibit compromised grana formation in a variety of plants including soybean^22^ and *Arabidopsis*^23^. In agreement with previous observations, the number of thylakoids per granum in transgenic lines was reduced in parallel with decreasing Chl *b* levels (Fig. 4a–h). Interestingly, the observed changes in thylakoid membrane structure in transgenic lines was not directly correlated with changes in Chl *a/b* ratios. Plants with slightly reduced Chl *b* levels (CR L-I) had slightly thicker double membranes and increased lumenal space compared to wild type (Fig. 4i–l). These less tightly appressed membranes structures in CR L-1 lines could contribute to improved photosynthesis rates since the greater lumenal volumes would facilitate diffusion of soluble electron transfer components such as plastocyanin and facilitate migration of light harvesting complexes during state-transitions or repair of damaged reaction center complexes. In contrast to WT and CR L-1 plants, CR H-I plants with Chl *a/b* ratios of 6–7 had thinner and more compact double thylakoid membrane which would impair diffusion of soluble and membrane associated proteins reducing electron transfer efficiency.

### Plant growth phenotype was related to the degree of antenna size reduction

**Figure 5:**
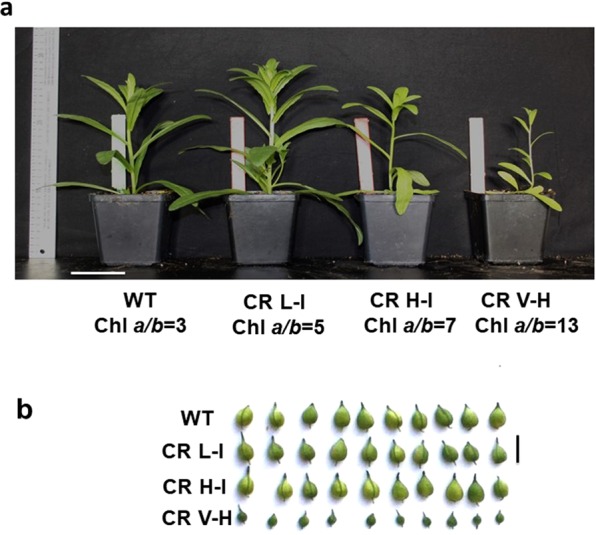
Growth phenotypes of WT and CR transgenic plants. (**a**) Comparison of growth phenotypes of 3-week old wild type (WT) and transgenic plants. Plants with low-intermediate antenna size (CR L-I) corresponding to Chl *a/b* ratios of 4–5 have more vigorous growth compared to both WT and plants with high-intermediate (CR H-I, Chl *a/b* ratios 6–8.5) and very high (CR V-H, Chl *a/b* ratios above 8.5)]. Scale bar, 10 cm. (**b**) Comparison of fully developed pod size in WT, CR L-I, CR H-I and CR V-H lines. The pod development in CR H-I and CR V-H lines is not compromised, while CR V-H pods are much smaller at maturity. Scale bar, 1 cm. Chl content from leaves was determined according to Arnon (1949)^33^.

Previous modeling studies suggest that a moderate increase in Chl *a/b* ratios or reductions in peripheral light harvesting antenna sizes can lead to increased photosynthetic performance and seasonal biomass accumulation^24^. We observed that plants having a slight reduction in antenna size (Chl *a/b* 5–6) demonstrated an increased early stage growth phenotype while plants with higher Chl *a/b* ratios were smaller than WT plants (Fig. 5a,b).

To determine if these differences in growth translated to the field, we carried out biomass and seed yield studies for WT and transgenic plants grown under field conditions (Supp. Fig. 4). The results of phenotypic analyses of field grown plants are shown in Table 1. Both WT and transgenic lines exhibited changes in Chl *a/b* values or antenna size between the top, middle, and bottom leaves. Notably, however, in all CR transgenic lines the Chl *a/b* ratios were greater at all levels in the canopy compared to corresponding levels in the canopy for wild-type plants. At the top of the canopy the Chl *a/b* ratios of WT plants were 3.2 +/− 0.1. For CR-LI, and CR H-1 plants the Chl *a/b* ratios were 5.0 +/− 0.3, and 7.0 +/− 0.4, respectively.

**Table 1:** Chl *a/b* ratios at different canopy levels of WT and CR L-I and two CR H-I lines grown in field studies.

Leaf position in canopy	WT Chl *a/b*	CR L-1 Chl *a/b*	CR H-1 Chl *a/b*
Top	3.2 ± 0.1	5.0 ± 0.3	7.0 ± 0.4
Middle	3.0 ± 0.1	4.3 ± 0.2	5.9 ± 0.4
Bottom	2.6 ± 0.2	3.9 ± 0.2	4.6 ± 0.3

Values shown are the means ± SE (10 plants).

Consistent with our previous observations on photosynthetic performance, there was a transition point at which increases in Chl *a/b* ratios or reductions in light harvesting antenna size stopped being beneficial for productivity. Overall, CR-LI transgenics having Chl *a/b* ratios (at the top of the canopy) near 5, had greater seed yield (+25%) and increases in total above ground biomass (+40%) compared to wild type than plants with higher Chl *a/b* ratios. Interestingly the increased seed yield was associated with greater branching, flowering and increased pod numbers in CR L-I plants (Table 2).

**Table 2:** Yield of WT and CR L-I and two CR H-I lines grown in field studies. nr = not relevant.

Traits	WT	CR L-I	CR H-I
Total seed weight (gdw/plant)	3.6 ± 0.4	4.5 ± 0.4	1.4 ± 0.1
Seed weight (gdw/plant) increase/decrease relative to WT (%)	nr	+25%	−61%
Above ground total plant dry weight (gdw/plant)	11.8 ± 1.3	17.0 ± 1.3	7.0 ± 0.5
Plant weight increase/decrease relative to WT (%)	nr	+44%	−40%
Number of seed pods per plant	481 ± 57	607 ± 53	292 ± 23
Seed pods/plant change relative to wild type	nr	+26%	-39%
Seed weight/pod (mgdw)	11.3 ± 0.6	10.0 ± 0.4	11.3 ± 0.6
Seed weight/pod change relative to wild type	nr	−12%	No change

Values shown are the means ± SE (30 plants).

We observed that even a slight increase in Chl *a/b* ratio greater than 5.0 resulted in an abrupt reduction in biomass productivity. The CR line having a Chl *a/b* value of 7.0, had a 61% decline in seed yield and 40% decline in biomass compared to WT (see Table 2).

## Discussion

At full sunlight intensities, plants absorb photons at a rate that exceeds the apparent rate limitations in photosynthetic electron transport resulting in the re-radiation of nearly 80% of the captured energy at full sunlight intensities. Furthermore, leaves having large antenna sizes shade or reduce light penetration into the canopy reducing photosynthetic rates in shaded leaves. Lowering leaf absorptivity to allow for greater canopy penetration would potentially increase light utilization efficiency throughout the plant canopy. Here, we describe the phenotypes of plants having a range of light harvesting antenna sizes. These studies revealed that plants having an upper canopy leaf Chl *a/b* ratio of near 5 had the highest photosynthetic and biomass production yields. Plants having Chl *a/b* ratios near 5 were shown to have lost approximately 1/3 of their LHCII trimer complexes. Unlike plants with higher Chl *a/b* ratios, plants with optimal Chl *a/b* ratios had higher photosynthetic rates, NPQ activity most similar to WT, and enhanced biomass productivity. Overall our results are similar to those observed for Chlorophytic algae (Chamydomonas) and plants engineered to have reduced antenna sizes^1,4,25,26^.

Significantly, reductions in peripheral light harvesting size associated with the loss of approximately one LHCII trimer complex results in increased photosynthetic and light stress response efficiencies, while additional reductions in antenna size actually decrease photosynthetic performance under stress. The transition point between more- and less-efficient photosynthesis corresponds to the elimination of higher-order PSII supercomplexes of photosystem II, C_2_S_2_M_2_, as well as one loosely attached L-trimers (CR L-1 has approximately 2/3 the LHC trimers of WT. By analyzing the thylakoid membranes of plants having various Chl *a/b* ratios, below and above this optimal antennae size transition point, we find that the thylakoid membranes of plants having Chl *a/b* ratios lower than the transition point (Chl *a/b* ratio ~ 5) have more tightly stacked thylakoids compared to wild-type and CR L-I plants, whereas plants with higher Chl *a/b* ratios had few and ballooned grana stacks. While it is not possible to resolve the arrangement of the individual complexes in thylakoid membranes by the techniques employed in this study, we speculate that the abrupt change in thylakoid stacking may represent a phase transition in the arrangement of PSII supercomplexes, with the equilibrium shifted towards a more crystalline structure in plants with antenna sizes associated with higher Chl *a/b* ratios (CR V-H). Highly ordered crystalline structures organized in regular 2D arrays observed by electron microscopy and atomic force microscopy studies are composed of unit cells containing one PSII core dimer and variable quantities of LHCII^27,28^. Thylakoid membrane phase transitions have previously been shown to be associated with alterations in the abundance of LHCII components^29^. In wild-type plants, the shift to a higher proportion of crystalline structure happens may result in reductions in photosynthetic efficiency in plants having very high Chl *a/b* ratios (CR V-H). Previous studies have shown that the formation of the PSII crystalline arrays in the Arabidopsis koLhcb6 mutant was associated with a lower photosynthetic electron transport rate^15^.

Under moderate and high light conditions efficient operation of the photosynthetic apparatus also requires fluidity of the thylakoid membrane structure^30^. This fluidity facilitates movement of highly mobile membrane protein complexes including migration of the cytochrome b6f complex; migration of LCHII complexes during NPQ; and the migration of PSII complexes to membrane margin regions for repair of damaged photosystem II complexes resulting from photoinhibition^31^. Therefore, the potential for antenna size modification to maximize photosynthetic performance is potentially constrained by the playoff between membrane crystallinity and fluidity as well as lumenal spacing facilitating diffusion of the electron carrier plastocyanin. Overall, these observations indicate that only narrow ranges of antenna size reduction can enhance photosynthetic performance through a multiplicity of effects not associated with antennae light harvesting cross section alone.

In summary, we engineered *Camelina sativa* plants with a range of Chl *a/b* ratios, resulting in a range of photosynthetic performance. Plants having small reductions in Chl *b* content had reduced peripheral light harvesting antenna corresponding to the loss of approximately one LHCII trimer complex. These plants had improved photosynthetic performance at high light intensities relative to WT as well as increased light penetration through the plant canopy allowing for increased total plant photosynthesis throughout the canopy. We have determined that there is a transition point where even a small change in Chl *a/b* values can result in significant differences in the composition of PSII supercomplexes and membrane architecture that can either enhance or reduce photosynthetic performance including organization of LHCII complexes, thylakoid membrane structure, and sensitivity to photoinhibition.

This raises the question; why have plants not optimized light harvesting efficiency. In nature evolution operates to maximize reproductive fitness. By shading competitors or limiting their access to light, there may well be a fitness advantage to having large, less efficient light harvesting antenna. In addition, large antenna allow for higher photosynthetic rate at very low light intensities allowing shaded plants to grow, but at a slower rate than at full sunlight intensities. In agricultural systems selection operates at the level of biomass yield and not necessarily reproductive fitness. Thus, enhancing yields through optimizing light use efficiency for a monocrop can take priority over reproductive fitness. Improvements in yield performance as described here when coupled with enhanced carbon reduction efficiency have the potential to substantially impact agriculture and food security.

## Materials and Methods

### Vector construction

The plasmid for RNAi–mediated silencing of the chlorophillide *a* oxygenase (CAO) gene was constructed using a genomic sense/cDNA anti-sense strategy. Since the sequence of the *Camelina sativa* CAO gene was unknown at the time, primers homologous to A. thaliana CAO sequence were used to amplify the CAO gene, *C. sativa* cDNA made from total RNA with qScript and the following forward (ATGAACGCCGCCGTGTTTAGT) and reverse (CGGTTCAGCGCAATGTCTCCA) were used for the PCR. The resulting PCR product was cloned by TA blunt cloning kit and six variants of the CAO gene were sequenced. The resulting sequences were used to design and synthesize the RNAi cassette under control of CAB1 leaf-specific promoter, which was placed in a modified pCambia1301 vector using EcoRI and HindIII restriction sites.

### Generation and screening or CAO RNAi transgenic lines

For the generation of CAO RNAi lines, we have transformed *Camelina sativa* using vacuum infiltration floral dip method^32^. The transformed plants were grown to maturity and the resulting seeds were screened on MS media (company) Agar plates supplemented with 25 ug/mL hygromycin as selection agent. The hygromycin-resistant seedlings were transferred to soil. The presence of the presence of RNAi sequence, the total plant DNA was extracted using a Qiagen kit and used as a PCR template. The presence of the transgene was confirmed by PCR using forward (5′) and reverse (3″) primers which hybridize to Cab1 promoter and Nos teminator sequences, respectively. Total plant DNA was isolated from leaves using an OMEGA kit (E.Z.N.A. Plant DNA DS Mini Kit) and used as a PCR template. PCR primers (forward, 5′-ATTGCGAATCTG CGAAGTGC-3′) corresponding to regions within the transgene promoter and (reverse, 5′-CTAGAGTCGACCTCGAGGGAT-3′) corresponding to regions within the CAO exon were used according to the manufacturer’s instructions. The PCR reaction was performed using the following conditions: 95 °C for 3 min to denature the DNA followed by 34 cycles of 94 °C (10 s), 58.9 °C (15 s), and 72 °C (20 s). PCR products of the predicted size were sequenced to confirm transgene identity.

### Plant materials and growth condition

Transgenic plants were selfed to generate T3 generation homozygous plants. Three to 9 week-old greenhouse grown plants were used for experiments. For most of the following experiments, fully expended top leaves were used. For leaf position dependency experiments, non-senescent fully mature leaves were used at the plant leaf position (from the top) noted.

### PCR verification of transgenics

Total plant DNA isolated from the leaves of WT and three transgenic lines was extracted using an OMEGA kit (E.Z.N.A. Plant DNA DS Mini Kit) and used as a PCR template. The presence of the CAO RNAi transgene was confirmed by PCR using forward (5′-ATTGCGAATCTG CGAAGTGC-3′) and reverse (5′-CTAGAGTCGACCTCGAGGGAT-3′) primers, according to the manufacturer’s instructions. PCR was performed under the following conditions: 95 °C for 3 min to denature the DNA followed by 34 cycles of 94 °C (10 s), 58.9 °C (15 s), and 72 °C (20 s).

### Chlorophyll determination

Camelina WT and CAO-RNAi plants were grown in greenhouse with a 14-h light /10-h dark rhythm. Fully expanded leaves of 3- to 5-week old plants were used in the experiments. Chl content from leaves was determined according to Arnon (1949)^33^ or Porra *et al*.^34^.

### Light transmittance though leaves

Light transmission through the leaves between wavelengths 400 nm and 700 nm was determined with BLACK-Comet CXR-SR-50 spectrometer (StellarNet Inc.). Full sunlight at midday was used as a light source. The experiment was repeated in triplicate using fully expanded leaves from the fifth position from the top of the plant.

### Photosynthetic CO_2_ fixation rate determination

Photosynthetic CO_2_ fixation rates were determined by gas-exchange measurements using a LI-6400 open-flow gas exchange system (Li-Cor). Photosynthetic light response curves were produced by increasing light intensity from 0 to 2000 μmol photons m^−2^ s^−1^. The reference CO_2_ concentration was set at 400 μmol CO_2_ mol^−1^ air. The leaf temperature was kept the 25 C and relative humidity at 50%. The measurements were done in triplicate on fully opened leaves (8–10th from the bottom) of 3.5 –week old plants.

### Chlorophyll fluorescence measurements

Chl *a* fluorescence was measured on detached leaved placed on wet filter paper using Handy FluorCam FC 1000-H (Photon Systems, Drasov, Chech Republic). Leaves were dark-adapted for 10 min, and minimal Chl fluorescence Fo was determined with low intensity measuring light pulses (620 nm). Then, a 0.8 s saturating pulse of white light (4,000 mmol photons m^−2^ s^−1^) was applied to determine the maximum fluorescence in a dark-adapted state, Fm. The leaves were then exposed to actinic white light at 100 umol m^−2^ s^−1^, for 60 sec, followed by 60 s of dark relaxation. Fm’, the maximum fluorescence in light-adapted state, was determined using a series of 0.8 s pulses of saturating white light. NPQ values were calculated as (Fm − Fm’)/Fm’.

### Isolation of thylakoid membranes

Isolation of thylakoid membranes was carried out according to Jarvi *et al*.^16^, with slight modifications. All steps were carried out in the green light at 4 °C. Fresh Camelina leaves were ground in a blender with ice cold grinding buffer (50 mM Hepes/KOH (pH 7.5), 330 mM sorbitol, 2 mM EDTA, 1 mM MgCl_2_, 5 mM ascorbate and 0.05% BSA and 1% SIGMAFAST Protease Inhibitor Tablets) and filtered through 2 layers of Miracloth. The suspension was briefly centrifuged at 500 g at 4 °C for 30 sec. The supernatant was centrifuged at 10,000 g for 10 min. The pellet was resuspended in a shock buffer [50 mM Hepes/KOH (pH 7.5), 5 mM sorbitol and 5 mM MgCl_2_ followed by centrifugation at 10,000 g at 4 °C for 10 min. Remnants of the shock buffer were removed by suspending the pellet into storage buffer [50 mM Hepes/KOH (pH 7.5), 100 mM sorbitol and 10 mM MgCl_2_ followed by centrifugation at 10000 g for 10 min. Finally, the thylakoid pellet was suspended into a small aliquot of storage buffer. The chlorophyll concentration was determined in aqueous 80% acetone according to Porra (1989)^34^.

### Blue-native gel electrophoresis

Blue-native gel electrophoresis was carried out according to Jarvi *et al*.^16^. Thylakoids containing 8 μg of Chl were solubilized for 10 min by addition of equal volume of buffer containing β-dodecyl-maltoside at a 2% w/v concentration. 1/10 volume of sample buffer containing Serva-Blue G was added and the sample was centrifuged in a microfuge for 10 min at maximum speed. Thylakoid complexes were resolved for 6-h at 4 °C in the dark on 4–12% Tris Tricine gel using Novex minigel system with a constant current of 6 mA. For the identification of LHCII trimers separated by SDGU, a 40 µL aliquot of LHCII trimer band was mixed with 4 µL sample buffer containing Serva-Blue G, centrifuged for 30 sec, and the supernatant was loaded on the gel for electrophoresis.

### Sucrose density gradient ultracentrifugation of detergent solubilized thylakoid chlorophyll protein complexes

Chlorophyll-protein complexes were isolated from detergent solubilized thylakoids by sucrose density gradient ultracentrifugation as described by Barera *et al*.^21^ with minor modifications. An aliquot of the thylakoid membrane extract containing 500 μg of chlorophyll was re-suspended for 1 min at 4°C in the dark in a buffer containing 25 mM MES (pH 6.5), 10 mM NaCl, 5 mM MgCl_2_ and 2 M glycine betaine, 2% α-DM. The solubilized fractions containing 400 μg of chlorophyll were loaded on the linear gradient of sucrose from 0.1 to 1.3 M and centrifuged at 100,000 g for 12 h at 4 °C, using a Superspin 630 rotor of WX Ultra 80 (Thermo Fisher Scientific).

### Sodium dodecyl sulfate polyacrylamide gel electrophoresis

Thylakoids containing 2 μg of Chl were solubilized for 30 min by addition of 20 μL of an SDS sample buffer containing 50mMTris-HCl (pH 6.8), 2% SDS, 0.1% bromophenol blue, 10% glycerol, and 1% β-mercaptoethanol at room temperature, and then, the sample was centrifuged in a microfuge for 10 min at maximum speed and the supernatant was loaded on a 10% (w/v) acrylamide gels. In the case of LHCII trimer band from BN-gel was solubilized for 60 min by addition of 1 mL of an SDS sample buffer and put on the acrylamide gels. And 2 μL LHCII trimers from SDGU were solubilized for 30 min by addition of 20 μL of an SDS sample buffer and loaded on the acrylamide gels. The electrophoresis was done for 2-h at 4 °C.

The solubilized samples were then loaded on 10% (w/v) acrylamide gels, and electrophoresis was performed at 80 V for 2 h at 4°C. Gels were stained in reagent made of 10% (v/v) acetic acid, 50% (v/v) methanol 40% (v/v) H_2_O, 0.1% (w/v) Coomassie Blue R250 for 1 h and washed with 10% acetic acid, 50% methanol, 40% (v/v) H_2_O at least 3 h.

### Electron microscopy

Camelina sativa leaves were collected at age 3 weeks and fixed in cacodylate buffer containing 2.5% glutaraldehyde. Post-fixation, embedding and sectioning were done as previously described (Rieder and Cassels, 1999)^35^. 100 nm-thin sections were imaged at Phillips/FEI T-12 microscope at 80 kV (sectioning and imaging were performed by Electron Microscopy Core of Vanderbilt University, Nashville, TN). ImageJ was used to measure thickness of thylakoid membranes and lumen. 5 chloroplasts sections were used for analysis for each sample for thylakoid membrane measurements, and 15 chloroplasts sections per sample were used for starch granules count.

### Field trials

The transgenic lines along with wild-type Camelina were planted at University of Nebraska Plant Biotechnology field facility in near Ithaca, Saunders County, Nebraska on April 16, 2015. The field is located at 41 o 08′N, 96 o 26′W. Seeds of the three transgenic lines and a wild type cultivar were planted in 2 meter long 12 × 12 row plots. The space between rows was 20 cm and the distance between each plot was 1 m. Seeds were carefully sown by hand uniformly to attain plant density of 450 per square meter. To ensure good plant establishment, plots received 6.5 mm irrigation five days after planting. No additional irrigation was needed due to sufficient rain throughout Camelina growth and development. Plots were fertilized with nitrogen in the form of urea at rate of 67 kg ha −1 on April 24, 2015. Depending on the need, weeding was performed by hand and plants were free of disease symptoms. To avoid border effects, plants in the outer three rows were excluded from data collection and analysis. Fully opened leaf samples from plants at the middle of the central rows were collected for chlorophyll analysis. Later, at physiological maturity, plants were cut at ground level to measure plant weight, seed weight, and number of pods per plant.

## Supplementary information

Supplementary information

## References

[1] Ort DR, Melis A (2011). Optimizing antenna size to maximize photosynthetic efficiency. Plant physiology.

[2] Subramanian S, Barry AN, Pieris S, Sayre RT (2013). Comparative energetics and kinetics of autotrophic lipid and starch metabolism in chlorophytic microalgae: implications for biomass and biofuel production. Biotechnology for biofuels.

[3] Beckmann J (2009). Improvement of light to biomass conversion by de-regulation of light-harvesting protein translation in Chlamydomonas reinhardtii. Journal of biotechnology.

[4] Perrine Z, Negi S, Sayre RT (2012). Optimization of photosynthetic light energy utilization by microalgae. Algal Research.

[5] Mussgnug JH (2007). Engineering photosynthetic light capture: impacts on improved solar energy to biomass conversion. Plant Biotechnology Journal.

[6] Hoober JK, Eggink LL, Chen M (2007). Chlorophylls, ligands and assembly of light-harvesting complexes in chloroplasts. Photosynthesis research.

[7] Eggink LL (2004). Synthesis of chlorophyll b: localization of chlorophyllide a oxygenase and discovery of a stable radical in the catalytic subunit. BMC Plant Biol.

[8] Ayumi Tanaka A (1998). Chlorophyll a oxygenase (CAO) is involved in chlorophyll b formation from chlorophyll a. Proc. Natl. Acad. Sci. USA.

[9] Kagale S (2014). The emerging biofuel crop Camelina sativa retains a highly undifferentiated hexaploid genome structure. *Nature*. Communications.

[10] Pettigrew WT (1989). Characterization of canopy photosynthesis of chlorophyll-deficient soybean isolines. Crop Science.

[11] Havaux M, Dall’osto L, Bassi R (2007). Zeaxanthin has enhanced antioxidant capacity with respect to all other xanthophylls in Arabidopsis leaves and functions independent of binding to PSII antennae. Plant Physiology.

[12] Zhu X-G, Song Q, Ort DR (2012). Elements of a dynamic systems model of canopy photosynthesis. Current opinion in plant biology..

[13] Ort DR (2015). Redesigning photosynthesis to sustainably meet global food and bioenergy demand. Proceedings of the National Academy of Sciences.

[14] Kovacs L (2006). Lack of the light-harvesting complex CP24 affects the structure and function of the grana membranes of higher plant chloroplasts. The Plant Cell.

[15] de Bianchi S, Dall’Osto L, Tognon G, Morosinotto T, Bassi R (2008). Minor antenna proteins CP24 and CP26 affect the interactions between photosystem II subunits and the electron transport rate in grana membranes of Arabidopsis. The Plant Cell.

[16] Järvi S, Suorsa M, Paakkarinen V, Aro E-M (2011). Optimized native gel systems for separation of thylakoid protein complexes: novel super-and mega-complexes. Biochemical Journal.

[17] Rantala M, Tikkanen M, Aro EM (2017). Proteomic characterization of hierarchical megacomplex formation in Arabidopsis thylakoid membrane. The Plant Journal.

[18] Caffarri S, Kouril R, Kereiche S, Boekema EJ, Croce R (2009). Functional architecture of higher plant photosystem II supercomplexes. The EMBO Journal.

[19] Boekema EJ, Van Roon H, Van Breemen JF, Dekker JP (1999). Supramolecular organization of photosystem II and its light-harvesting antenna in partially solubilized photosystem II membranes. European Journal of Biochemistry/FEBS.

[20] Dekker JP, Boekema EJ (2005). Supramolecular organization of thylakoid membrane proteins in green plants. Biochimica et Biophysica Acta (BBA)-Bioenergetics.

[21] Barera S, Pagliano C, Pape T, Saracco G, Barber J (2012). Characterization of PSII–LHCII supercomplexes isolated from pea thylakoid membrane by one-step treatment with α-and β-dodecyl-d-maltoside. Philosophical Transactions of the Royal Society B: Biological Sciences.

[22] Nakanishi H (2005). Characterization of the Arabidopsis thaliana mutant pcb2 which accumulates divinyl chlorophylls. Plant & Cell Physiology.

[23] Keck RW, Dilley RA, Allen CF, Biggs S (1970). Chloroplast composition and structure differences in a soybean mutant. Plant Physiology.

[24] Kirst H, García-Cerdán JG, Zurbriggen A, Melis A (2012). Assembly of the light-harvesting chlorophyll antenna in the green alga Chlamydomonas reinhardtii requires expression of the TLA2-CpFTSY gene. Plant Physiology.

[25] Kirst H, Melis A (2014). The chloroplast signal recognition particle (CpSRP) pathway as a tool to minimize chlorophyll antenna size and maximize photosynthetic productivity. Biotechnology Advances.

[26] Pettigrew W, Turley R (1998). Variation in photosynthetic components among photosynthetically diverse cotton genotypes. Photosynthesis Research.

[27] Kouřil R, Dekker JP, Boekema EJ (2012). Supramolecular organization of photosystem II in green plants. Biochimica et Biophysica Acta (BBA)-Bioenergetics.

[28] Hankamer B, Barber J, Boekema EJ (1997). Structure and membrane organization of photosystem II in green plants. Annual Review of Plant Biology.

[29] Schneider AR, Geissler PL (2013). Coexistence of fluid and crystalline phases of proteins in photosynthetic membranes. Biophysical Journal.

[30] Yamamoto Y (2014). Quality control of PSII: behavior of PSII in the highly crowded grana thylakoids under excessive light. Plant & Cell Physiology.

[31] Kouril R, Wientjes E, Bultema JB, Croce R, Boekema EJ (2013). High-light vs. low-light: effect of light acclimation on photosystem II composition and organization in Arabidopsis thaliana. Biochimica et Biophysica Acta.

[32] Clough SJ, Bent AF (1998). Floral dip: a simplified method for Agrobacterium‐mediated transformation of Arabidopsis thaliana. The Plant Journal.

[33] Arnon DI (1949). Copper enzymes in isolated chloroplasts. Polyphenoloxidase in Beta vulgaris. Plant Physiology.

[34] Porra R, Thompson W, Kriedemann P (1989). Determination of accurate extinction coefficients and simultaneous equations for assaying chlorophylls a and b extracted with four different solvents: verification of the concentration of chlorophyll standards by atomic absorption spectroscopy. Biochimica et Biophysica Acta (BBA)-Bioenergetics.

[35] Rieder, C. L. & Cassels, G. In *Methods in cell biology*, Vol. 61, 297–315 (Elsevier, 1998). 10.1016/s0091-679x(08)61987-1 9891321

