# Supplementary material for: Fine-tuning the photosynthetic light harvesting apparatus for improved photosynthetic efficiency and biomass yield
Source: Sci Rep. 2019 Sep 10;9:13028. doi: 10.1038/s41598-019-49545-8 (PMC6736957; doi:10.1038/s41598-019-49545-8)
Supplement: Supplementary file 1 — Supplementary information [file 41598_2019_49545_MOESM1_ESM.pdf]

Supplementary Information

Fine-tuning the photosynthetic light harvesting apparatus for improved photosynthetic efficiency and biomass yield

Friedland N[1], Negi S[1], Vinogradova-Shah T[1,2], Wu G[1,3] Ma L[1,3,], Flynn S[1], Kumssa T[4], Lee C-H[1,3], and Sayre RT[1,2]*

[1]New Mexico Consortium, Los Alamos, NM
[2]Pebble Labs, 100 Entrada Drive, Los Alamos, NM
[3]Department of Molecular Biology, Pusan National University, Busan 46241 Republic of Korea
[4]University of Nebraska, Lincoln, NE

Table of Contents:

Page 3. Supplementary Figure S1: DNA sequence of CAO RNAi construct used to down-regulate the expression of the native *C. sativa* CAO gene.

Page 4. Supplementary Figure S2. a) Light transmission though leaves of wild type and transgenic plants. b) Ten week old CRL-I and wild type plants grown in the green house.

Page 5. Supplementary Figure S3. Blue native gel analysis of LHCII trimer fraction isolated by SDGU.

Page 6. Supplementary figure S4. Field study planting site in in Ithaca, Saunders County, Nebraska.

Page 7. Supplementary Table S1. Leaf position dependency of Chl *a/b* ratio in wild-type and CR transgenic plants

Page 8. Supplementary Table S2. Chlorophyll content/per and Chl *a/b* ratio of an equal unit volume of LHCII trimer complexes from wild-type and CR transgenic lines

Supplementary Figure S1

atgaacgccgccgtgtttagtgcttctgctttatctcttcctatctccttctctaaaaccagatcatcttatctctctagaaagaagggagtgaaaggagagttcagggtttat
gctgtgtttggtgatgagagtggattagttgagaagaagagtcagtggagacctttgtttgatgttgaggatcctagatcaaaagctcctccttataaaggcaagttcttgg
atgttaaccaagcgattgaagttgctagattcgatattcaatacttggattggcgtgctcgtcaagatcttcttaccattatgattcttcatgacaaggtttgtttctacattgat
ctcttttgaacttgtttatgtctagttagttggtaaagttgtgttttatcgagttgtagttaaggacatagctataatatgctacatttagttctagggtatatatagtagacgta
tagctatgactctagtagctcaagttcttcaaatgttgtttttgtagcactctttatagtattgacatttgacaacccattaatggtttatgtttaagtgttttgatctaagtttgt
ggaatgaatgtatgttaagggtagatgtactaaatcccctagctcgtgagtacaagtccatcggtactgtgaagaaagaactagctggattgcaggaagaactatcaaa
agcacaccaacaggttcatatatctgaagcaagggtttcaactgctttagacaagttagctcacatggaagaactggtaaatgataggttgttacctggcagagttgtaac
ggatttagataaaccctcctcttcaacctctacttctactatcgagttagacagggttaacaatgctattcactgagaatgattagtttttttatgagacttcctctaactctta
gttgaaatcaattggcaccattgtgtcatgcttgagatctgcagtgaaagcgaccggataccaaaaattcttcaagtttggactataaggcggaaccggaccagaaaca
ttcaagctcttagcaccagtgtttgtcttttccctgtctaactcgatagtagaagtagaggttgaagaggagggtttatctaaatccgttacaactctgccaggtaacaacct
atcatttaccagttcttccatgtgagctaacttgtctaaagcagttgaaacccttgcttcagatatatgaacctgaacctgttggtgtgcttttgatagttcttcctgcaatcca
gctagttctttcttcacagtaccgatggacttgtactcacgagctaggggatttagtacatcaccttgtcatgaagaatcataatggtaagaagatcttgacgagcacgcca
atccaagtattgaatatcgaatctagcaacttcaatcgcttggttaacatccaagaacttgcctttataaggaggagcttttgatctaggatcctcaacatcaaacaaaggt
ctccactgactcttcttctcaactaatccactctcatcaccaaacacagcataaaccctgaactctcctttcactcccttctttctagagagataagatgatctggttttagag
aaggagataggaagagataaagcagaagcactaaacacggcggcgttcataagcttcat

DNA sequence of CAO RNAi construct used to down-regulate the expression of the native *C. sativa* CAO gene. The sequence contains two exon sequences from *Camelina* CAO gene (shown in red) interspersed with two intron sequences from CAO gene of *A. thaliana* (shown in black), followed by the reverse complemented sequence of the two exons (shown in blue).

Supplementary Figure S2

a

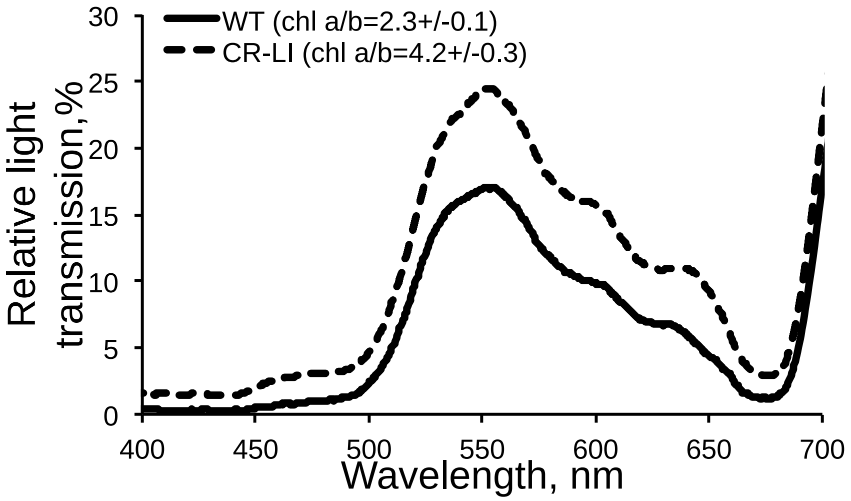

b

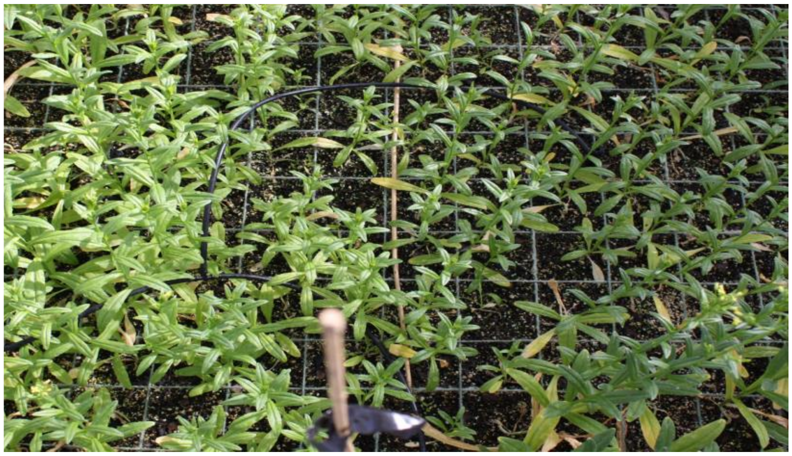

CR L-I
Chl a/b=5-6

WT
Chl a/b=3

(a) **Light transmission though leaves of wild-type and transgenic plants**. The transmission spectra were normalized to the total incident sunlight and taken from the fifth, fully expanded, leaf from the apex Data presented is the average of three independent plant measurements

(b) **Ten week old CRL-I and wild-type plants grown in the green house.** Plant densities are similar to those used in the field. Note the differences in leaf color and relative plant height between CR L-1 and wild-type (WT) plants.

Supplementary Figure S3

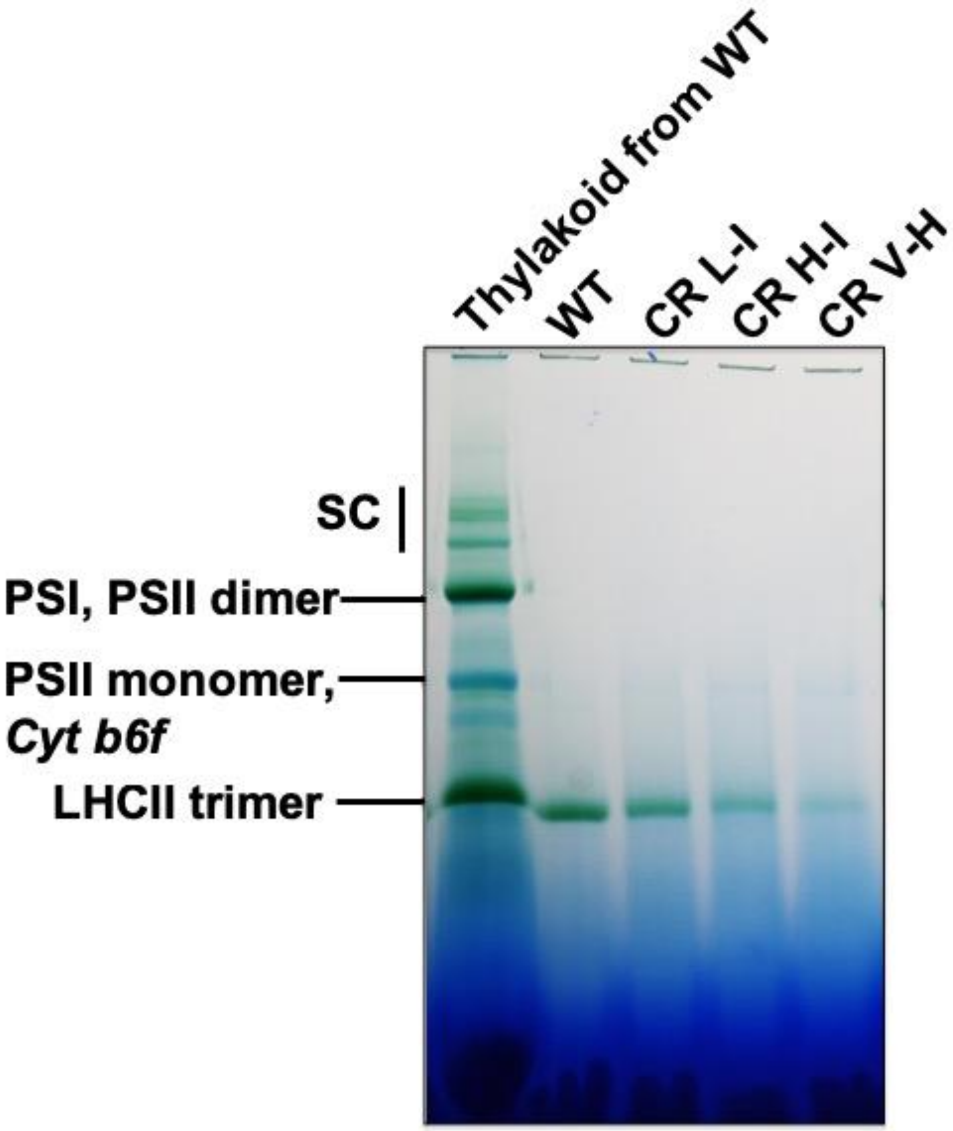

Blue native gel analysis of LHCII trimer fraction isolated by SDGU. Identical volumes of LHCII trimer bands from SDGU were resolved on 4%-16% BN-gel. SC is supercomplexes.

Supplementary Figure S4

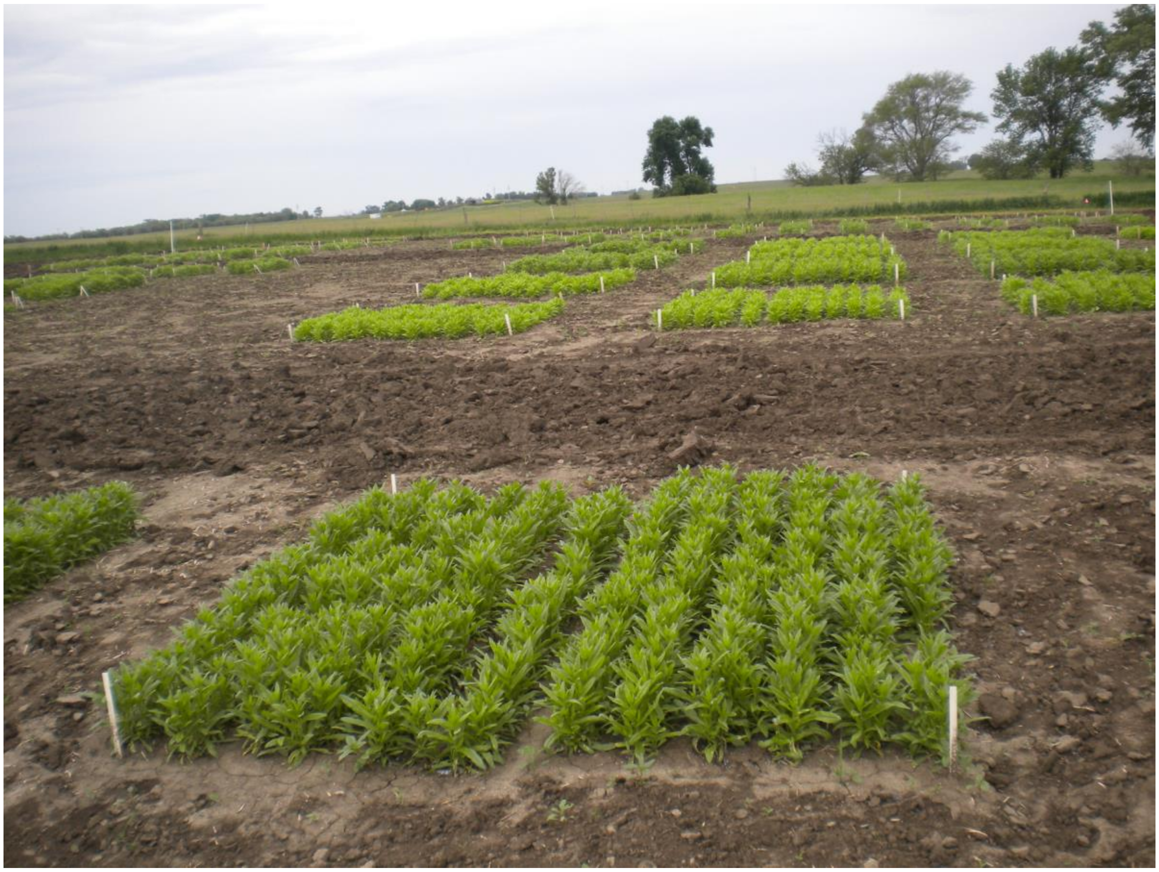

Field study planting site in in Ithaca, Saunders County, Nebraska

Supplementary Table S1

Sample	Bottom Leaf	Middle Leaf	Top Leaf
**WT - 5 weeks old**	3.47 ± 0.11	3.89 ± 0.12	4.16 ± 0.10
**WT – 7 weeks old**	3.17 ± 0.06	3.87 ± o.10	4.09 ± 0.10
**CR L-1 – 5 weeks old**	5.85 ± 1.39	6.25 ± 1.23	6.46 ± 0.56
**CR L-1 – 7 weeks old**	4.55 ± 0.66	5.16 ± 0.33	5.89 ± 0.25
**CR H-1 – 5 weeks old**	7.07 ± 1.65	8.45 ± 0.16	8.22 ± 0.84
**CR H-1 – 7 weeks old**	7.72 ± 0.68	8.23 ± 0.09	8.98 ± 0.54
**CR V-H - 5 weeks old**	8.37 ± 0.49	10.27 ± 1.37	9.24 ± 1.00
**CR V-H - 7 weeks old**	11.11 ± 2.67	11.02 ± 1.58	10.87 ± 1.85

Leaf position dependency of Chl *a/b* ratio in wild-type and CR transgenic plants. Fully expended non-senescent leaves at the bottom, middle and top position of plants of plants ranging from 5 and 7 week-old age were compared. Results are the average and ±SD of at least 3 independent experiments. Chl content from leaves was determined according to Porra *et al*. (1989)[24]

Supplementary Table S2

Sample	*Chl a + b* (µg/unit volume)	*Chl a/b* ratio
**WT**	92.17 ± 7.58<br>100%	1.56 ± 0.10
**CR L-1**	72.86 ± 12.51<br>79%	2.02 ± 0.24
**CR H-1**	57.21 ± 6.22<br>62%	2.20 ± 0.24
**CR V-H**	41.98 ± 4.24<br>46%	4.45 ± 0.77

Chlorophyll content/per and Chl *a/b* ratio of an equal unit volume of LHCII trimer complexes from wild-type and CR transgenic lines. Chlorophyll analyses were made using isolate LHCII trimer fractions from SDGU as described in Fig. 3. Results are the average and ±SD of at least 3 independent experiments. Data is expressed as a percentage of wild type (100%). Chl content was determined according to Porra *et al.* (1989)[24]
